# Carbon trading, co-pollutants, and environmental equity: Evidence from California’s cap-and-trade program (2011–2015)

**DOI:** 10.1371/journal.pmed.1002604

**Published:** 2018-07-10

**Authors:** Lara Cushing, Dan Blaustein-Rejto, Madeline Wander, Manuel Pastor, James Sadd, Allen Zhu, Rachel Morello-Frosch

**Affiliations:** 1 Department of Health Education, San Francisco State University, San Francisco, California, United States of America; 2 Department of Environmental Science, Policy and Management, University of California, Berkeley, Berkeley, California, United States of America; 3 Goldman School of Public Policy, University of California, Berkeley, Berkeley, California, United States of America; 4 Program for Environmental and Regional Equity, University of Southern California, Los Angeles, California, United States of America; 5 Department of Geology, Occidental College, Los Angeles, California, United States of America; 6 Department of Electrical Engineering and Computer Sciences, University of California, Berkeley, Berkeley, California, United States of America; 7 School of Public Health, University of California, Berkeley, Berkeley, California, United States of America; University of Wisconsin, Madison, UNITED STATES

## Abstract

**Background:**

Policies to mitigate climate change by reducing greenhouse gas (GHG) emissions can yield public health benefits by also reducing emissions of hazardous co-pollutants, such as air toxics and particulate matter. Socioeconomically disadvantaged communities are typically disproportionately exposed to air pollutants, and therefore climate policy could also potentially reduce these environmental inequities. We sought to explore potential social disparities in GHG and co-pollutant emissions under an existing carbon trading program—the dominant approach to GHG regulation in the US and globally.

**Methods and findings:**

We examined the relationship between multiple measures of neighborhood disadvantage and the location of GHG and co-pollutant emissions from facilities regulated under California’s cap-and-trade program—the world’s fourth largest operational carbon trading program. We examined temporal patterns in annual average emissions of GHGs, particulate matter (PM_2.5_), nitrogen oxides, sulfur oxides, volatile organic compounds, and air toxics before (January 1, 2011–December 31, 2012) and after (January 1, 2013–December 31, 2015) the initiation of carbon trading. We found that facilities regulated under California’s cap-and-trade program are disproportionately located in economically disadvantaged neighborhoods with higher proportions of residents of color, and that the quantities of co-pollutant emissions from these facilities were correlated with GHG emissions through time. Moreover, the majority (52%) of regulated facilities reported higher annual average local (in-state) GHG emissions since the initiation of trading. Neighborhoods that experienced increases in annual average GHG and co-pollutant emissions from regulated facilities nearby after trading began had higher proportions of people of color and poor, less educated, and linguistically isolated residents, compared to neighborhoods that experienced decreases in GHGs. These study results reflect preliminary emissions and social equity patterns of the first 3 years of California’s cap-and-trade program for which data are available. Due to data limitations, this analysis did not assess the emissions and equity implications of GHG reductions from transportation-related emission sources. Future emission patterns may shift, due to changes in industrial production decisions and policy initiatives that further incentivize local GHG and co-pollutant reductions in disadvantaged communities.

**Conclusions:**

To our knowledge, this is the first study to examine social disparities in GHG and co-pollutant emissions under an existing carbon trading program. Our results indicate that, thus far, California’s cap-and-trade program has not yielded improvements in environmental equity with respect to health-damaging co-pollutant emissions. This could change, however, as the cap on GHG emissions is gradually lowered in the future. The incorporation of additional policy and regulatory elements that incentivize more local emission reductions in disadvantaged communities could enhance the local air quality and environmental equity benefits of California’s climate change mitigation efforts.

## Abstract 2

Rachel Morello-Frosch and colleagues reveal the health inequities in California’s carbon trading program with a disproportionate number of greenhouse gas-regulated facilities in economically disadvantaged neighbourhoods reported.

## Author summary

**Why was this study done?:**

- Climate change policies to reduce greenhouse gas (GHG) emissions can also reduce emissions of hazardous co-pollutants, such as air toxics and particulate matter.
- Decreases in GHG emissions are therefore also likely to provide health benefits by improving local air quality to communities near regulated facilities.
- Globally, socioeconomically disadvantaged communities are often disproportionately exposed to hazardous air pollutants due to emissions from facilities nearby.
- We examined temporal patterns in GHG and co-pollutant emissions with respect to neighborhood demographics under California’s cap-and-trade program—the world’s fourth largest carbon trading market.

**What did the researchers do and find?:**

- We assessed GHG and co-pollutant (particulate matter, nitrogen oxides, sulfur oxides, volatile organic compounds, and air toxics) emission patterns and the social equity implications of California’s cap-and-trade program before (2011–2012) and after (2013–2015) the initiation of carbon trading.
- Facilities regulated under California’s cap-and-trade program are disproportionately located in disadvantaged neighborhoods.
- Statistical analysis found that co-pollutant emissions from regulated facilities were temporally correlated with GHG emissions, and most regulated facilities (52%) reported higher annual average local (in-state) GHG emissions after the initiation of trading, even though total emissions remained well under the cap established by the program.
- Since California’s cap-and-trade program began, neighborhoods that experienced increases in annual average GHG and co-pollutant emissions from regulated facilities nearby had higher proportions of people of color and poor, less educated, and linguistically isolated residents, compared to neighborhoods that experienced decreases in GHGs.

**What do these findings mean?:**

- To our knowledge, this is the first study to assess social disparities in GHG and co-pollutant emissions under an existing carbon trading program.
- Although GHG emission reductions could bring about significant air quality and health benefits for California’s disadvantaged residents, thus far the state’s cap-and-trade program has yet to yield such localized improvements in environmental equity.
- Policy and regulatory incentives to enhance local GHG emission reductions in disadvantaged communities could yield greater local air quality and environmental equity benefits from California’s climate change mitigation strategies.
- Future regulatory efforts should systematically track trends in hazardous co-pollutant emissions associated with GHG emissions from stationary and transportation-related sources and assess how they impact socioeconomically disadvantaged populations.

## Introduction

### Health and environmental equity benefits of reducing greenhouse gases (GHGs)

GHGs, including carbon dioxide (CO_2_), indirectly impact health by causing climate change but are not directly harmful at the concentrations typically found in outdoor air. However, GHG emissions from the combustion of fossil fuels are accompanied by other hazardous co-pollutants such as particulate matter (PM), ozone-forming nitrogen oxides (NO_x_), and volatile organic compounds (VOCs) that cause respiratory and cardiovascular disease and increases in mortality [1]. Decreases in GHG emissions from combustion are thus likely to provide short- and long-term health benefits by improving local air quality [2]. Several studies estimate that the economic cost savings of reduced air-pollution-related illness and death often outweigh the costs of GHG mitigation [3–5].

Globally, socioeconomically disadvantaged communities are often disproportionately exposed to hazardous air pollutants [6,7]. In the US, regulation under the Clean Air Act has led to significant improvements in ambient air quality even while the economy and population have grown [8]. However, many air toxics remain unregulated, and some industrial facilities are exempt from regulation due to their vintage, size, or location. Moreover, many US cities are out of compliance with ambient air quality standards, and stark racial, ethnic, and class-based inequalities in exposure to air pollutants remain [9]. For example, in the US, Asian American, African American, and Hispanic individuals (herein referred to as “people of color”) have higher estimated lifetime cancer risks from exposure to hazardous ambient air pollutants compared to white individuals [10]. Similarly, based on location of residence, average outdoor nitrogen dioxide levels are 38% higher for people of color than for non-Hispanic white individuals, and reducing ambient concentrations to the level experienced by white individuals would reduce ischemic heart disease mortality by an estimated 7,000 deaths per year [11].

Strategies to reduce GHG emissions could be structured to also maximize the ancillary health benefits of reducing these social inequalities in exposure to air pollutants that have persisted despite decades of regulation. Indeed, emerging evidence suggests that designing air quality regulations to improve conditions for those who are most negatively impacted can also efficiently improve overall outcomes at the population level. For example, Levy et al. examined the equity and efficiency benefits of a suite of hypothetical rollouts of emission-control technology at US power plants, by simulating scenarios by which reductions of sulfur dioxide (SO_2_), NO_x_, and fine PM (PM_2.5_) could be distributed to achieve national emission caps. The authors applied a source–receptor matrix to determine pollutant concentration changes associated with various control scenarios and mortality reductions, and estimated changes in the spatial inequality of health risk, applying the Atkinson index for health risk inequality. Study results found that reductions in spatial inequality in mortality associated with SO_2_ and PM_2.5_ emissions were correlated with higher total mortality reductions [12]. A later study looking at controls on tail-pipe emissions on public buses in Boston, using a similar source–receptor matrix method and inequality metric, found similar results [13].

### Environmental equity concerns regarding cap-and-trade

Cap-and-trade has emerged as the dominant regulatory mechanism for pricing carbon and reducing GHG emissions from large stationary sources around the world. Under a cap-and-trade system, regulated companies must surrender tradable emission permits, or “allowances,” equal to the amount of GHGs they emit (typically, 1 allowance equals 1,000 kg [1 metric ton (t)] CO_2_ equivalent [CO_2e_]). The cap on emissions is set by the total allowances issued, which is designed to decrease over time to secure aggregate gains. As the cap is lowered, regulated companies can reduce their GHG emissions (e.g., through energy efficiency measures, new technologies, or switching to less GHG-intensive fuels) or purchase excess allowances from other regulated entities that are able to reduce their emissions more cheaply. Most cap-and-trade programs also allow industries to purchase carbon offset credits generated from projects in sectors outside of the cap and often outside of the legal jurisdiction of the program—such as forestry or agriculture projects in other states or countries—that can be used in place of allowances. The market-based approach of cap-and-trade ostensibly lowers emission reduction costs and enhances industry support for climate change mitigation policies [14].

Some economists and environmental justice advocates argue that efficient climate regulation requires deeper GHG reductions in locations where the health benefits of co-pollutant reductions are likely to be greatest, and that this objective cannot be accomplished with the geographically unrestricted trading characteristic of cap-and-trade in which all GHG reductions are treated equally regardless of where they occur [15]. Offsets may further undermine improvements to local air pollution by undercutting financial incentives for industries to reduce emissions on site. Unless the location and co-pollutant intensity of GHG emissions are incorporated into the design of a cap-and-trade system, carbon trading could also potentially widen social inequities in exposure to localized hazardous co-pollutants because GHG-emitting facilities, which are disproportionately located in disadvantaged communities, are able to purchase allowances or offsets rather than reduce their emissions [15–17]. However, to our knowledge, no studies have examined trends in co-pollutant emissions or social disparities in emission reductions under an existing carbon trading program in order to inform climate policy design.

## Methods

Using data from January 1, 2011–December 31, 2015, which includes the first 3 years of California’s cap-and-trade program, we evaluated temporal and sector-specific trends in emissions of GHGs and hazardous co-pollutants overall and with respect to multiple measures of neighborhood demographics and disadvantage. Specifically, our analysis sought to examine the following questions: (1) What are the demographic characteristics of neighborhoods (census block groups) surrounding facilities that are currently regulated under California’s cap-and-trade program? (2) Since the program’s implementation, what patterns are evident in terms of the relationship between local (in-state) GHG and co-pollutant emissions across and between industry sectors? (3) What is the relationship between neighborhood demographics and temporal patterns in local GHG and co-pollutant emissions? (4) What trends do we observe in terms of companies that utilize offsets as part of their regulatory compliance obligations and their local emissions of GHGs and co-pollutants?

### California’s cap-and-trade program

California’s cap-and-trade program regulates carbon dioxide, methane, nitrous oxide, and fluorinated GHGs from power plants, refineries, industrial facilities, fuel suppliers, and other entities that emit over 25,000 t CO_2e_ of GHGs per year, with biogenic CO_2_ being exempt. The program covers 3 types of GHG emissions: (1) direct emissions within the state (“local” emissions); (2) indirect emissions from electricity imported from outside state boundaries; and (3) starting in 2015, geographically distributed emissions from fuels such as gasoline and natural gas. Beginning in 2013, industries were required to hold allowances equal to their GHG emissions (1 allowance = 1 t CO_2e_). Over 90% of allowances were freely allocated during the first compliance period of 2013–2014, with the balance auctioned or reserved for price containment. The total number of allowances in circulation, or “cap,” decreases by 3%–3.5% annually between 2015 and 2020 in order to meet a cumulative GHG reduction target of 15% from 2015 to 2020. In addition, companies can meet 8% of their compliance obligation by purchasing GHG emission reduction credits generated by offset projects located in the US (1 offset = 1 t CO_2e_). Thus, by design, the 3%–3.5% annual reduction in GHG emissions set by the decreasing cap can be achieved entirely via offset projects. Cutbacks in the use of more carbon intensive energy sources imported from outside the state (such as electricity generated from coal-fired rather than natural gas power plants) can also be used by regulated entities to meet emission reduction goals in lieu of in-state reductions.

Recognizing the social and environmental equity concerns related to cap-and-trade, California passed legislation requiring that 25% of the revenue generated by the program be invested in climate mitigation measures located in or benefitting disadvantaged communities [18]. These communities are defined geographically based on CalEnviroScreen, a spatial mapping tool that combines 21 indicators of environmental quality and population vulnerability to identify communities most burdened by multiple sources of pollution and that may be especially vulnerable to their effects [19]. CalEnviroScreen incorporates measures of ambient pollution and proximity to pollution sources, most of which are not regulated under cap-and-trade; these measures include hazardous waste sites, polluted water bodies, traffic density, pesticide usage, drinking water quality, and ambient air quality measures for ozone and PM_2.5_. CalEnviroScreen also includes indicators of population vulnerability including low educational attainment, poverty, linguistic isolation, and unemployment, and measures of health status, because of the evidence that social stressors and underlying chronic health conditions may exacerbate the adverse effects of pollution exposures [20]. A recent systematic review of relevant human and animal studies using the Navigation Guide protocol [21] assessed the combined impact and interaction of prenatal exposure to stressors and chemicals, including air pollution, on developmental outcomes. For the most common outcome (fetal growth), the authors evaluated risk of bias, calculated effect sizes for main effects of individual and combined exposures, and found that, in human studies, effect estimates for pollutants were stronger for groups exposed to higher levels of social stressors [22].

We utilize neighborhood demographic measures from the US Census and the CalEnviroScreen designation of “disadvantaged communities”—which are the 25% of California census tracts that score the worst on measures of environmental quality and population vulnerability—to analyze the distribution of GHG and co-pollutant emissions from facilities regulated under California’s cap-and-trade program.

### Emissions data

GHG and co-pollutant emissions from facilities regulated under California’s cap-and-trade program were downloaded from the Pollution Mapping Tool (formerly known as the Integrated Emissions Visualization Tool) of the California Air Resources Board (CARB) for the calendar years 2011–2015 (https://www.arb.ca.gov/ei/tools/pollution_map/). The locations (latitude and longitude) of facilities were obtained separately from CARB and were based on geo-coding of facility-reported addresses. The locational information was manually cleaned using satellite imagery from Google Earth to verify the location of GHG facilities. Facility-level GHG emissions are self-reported to the State of California under the Regulation for the Mandatory Reporting of Greenhouse Gas Emissions (mandatory reporting regulation [MRR]) program [23] and include self-reported estimates of annual carbon dioxide (CO_2_), methane (CH_4_), nitrous oxide (N_2_O), and fluorinated GHG emissions that have been verified by an independent third party. Our analysis focused on “emitter covered” emissions (local emissions), which correspond to localized, in-state emissions resulting from “the combustion of fossil fuels, chemical and physical processes, vented emissions,” and “emissions from suppliers of carbon dioxide” as well as emissions of GHGs other than CO_2_ from biogenic fuel combustion. Emissions are given in units of CO_2e_ based on the 100-year global warming potential factors given in Title 40, Part 98, of the Code of Federal Regulations, Subpart A, Table A-1, as published in the Federal Register on October 30, 2009 (http://www.arb.ca.gov/cc/reporting/ghg-rep/regulation/subpart_a_rule_part98.pdf).

Facility-level emissions of PM, NO_x_, sulfur oxides (SO_x_), and VOCs are self-reported by regulated facilities under the California Emission Inventory Development and Reporting System (CEIDARS) program. Reporting under CEIDARS is required every 4 years but may be more frequent depending on the administrative air basin and facility. We made several adjustments to harmonize the MRR and CEIDARS datasets. First, the GHG emissions for 3 hydrogen plants were allocated to nearby refineries because they primarily produce hydrogen for those refineries and because the facilities appear to report jointly to CEIDARS. Second, we summed co-pollutant emissions from oil and gas facilities based on a cross-walk file provided by CARB in order to harmonize the data with oil and gas GHG emissions that are reported on a more aggregated basis to the MRR. Finally, 4 facilities merged into or were acquired by 2 other facilities during the study period. Emissions prior to the merger for these facilities were combined for consistency in temporal reporting.

Data on the annual stack emissions of air toxics were downloaded from the US Environmental Protection Agency’s Risk-Screening Environmental Indicators (RSEI) model using the EasyRSEI application and matched to regulated California GHG facilities based on their name and spatial proximity (https://www.epa.gov/rsei). In a few cases, if the facilities in the RSEI and MRR databases were near each other but the names did not match, an internet search was used to confirm that one company was a subsidiary of the other. RSEI emission estimates come from data that are self-reported to the Toxics Release Inventory (TRI) as required by Section 313 of the Emergency Planning and Community Right-to-Know-Act of 1986 [24]. Facilities must report to the TRI if they are in a specific industry sector (such as mining, utilities, manufacturing, and hazardous waste facilities), employ 10 or more full-time employees, and manufacture, process, or handle a TRI-listed chemical in sufficient quantities (https://www.epa.gov/toxics-release-inventory-tri-program/basics-tri-reporting). Only a fraction of facilities regulated under California’s cap-and-trade program are required to report to TRI.

Facilities were initially categorized according to the first 2 digits of the North American Industry Classification System (NAICS) codes given in the MRR. In order to facilitate the analysis, we then grouped several categories together to achieve a greater number of facilities in each category as follows: educational, healthcare and social assistance, professional, scientific, technical, public administration, and other services were categorized as “public services”; mining, quarrying, and oil and gas extraction were categorized as “oil and gas production/supplier”; facilities in the “utilities” category with a NAICS description of “steam and air-conditioning supply” were grouped as “co-generation”; support activities for transportation, agriculture, forestry, fishing and hunting, utilities, arts, entertainment, recreation, information, wholesale trade, administrative and support, and waste management and remediation services, and missing values were recoded as “other.” The category of paper, chemical, mineral, and petroleum manufacturing was renamed “other manufacturing.” All other categories are as coded by NAICS.

Finally, we conducted several additional data cleaning steps. We concluded from a visual inspection of the 43 facilities that reported 0 emitter covered (i.e., local) GHG emissions during 1 or more years that many of these 0 values were likely not true 0s, but artifacts of the accounting rules that govern which emissions are covered under the program. Most facilities reporting 0 emitter covered GHG emissions reported total GHG emissions during the same year, and had reported emitter covered GHG emissions proportional to their total GHG emissions in all other years. Therefore, we replaced 0 emitter covered GHG values with the value of total GHGs reported by that facility during the same year, multiplied by the ratio of emitter covered to total GHGs reported the prior year, or the subsequent year if the prior year was not available. Finally, 5 GHG facilities reported 0 GHG emissions and then stopped reporting in subsequent years. We assumed these facilities ceased operations during the study period and assumed 0 GHG emissions for all years after reporting ceased.

### Neighborhood demographics

We defined neighborhoods on the basis of 2010 vintage census block group boundaries provided by the US Census Bureau (https://www.census.gov/geo/maps-data/data/cbf/cbf_blkgrp.html). Block groups are generally contiguous geographic areas that contain between 600 and 3,000 people and can vary in size depending on population density. Geographic block group centroids and the distance between block group centroids and GHG facility locations were calculated using ArcGIS (ESRI, Redlands, CA). We considered 2 buffer distances when assigning block groups to nearby GHG facilities based on their geographic centroid: 1 mile (1.6 km) and 2.5 miles (4.0 km).

Demographic information for each block group was obtained from the American Community Survey 2011–2015 5-year estimates (https://www.census.gov/acs/www/). White individuals were defined as those who self-identified as white race but not Hispanic ethnicity. People of color were defined as all other individuals, including those who identified as multiracial or of Hispanic ethnicity. Poverty was defined as twice the federal poverty level to reflect increases in the cost of living and California’s high cost of living relative to the rest of the country [25]. CalEnviroScreen 3.0 scores for all census tracts were obtained from the California Office of Environmental Health Hazard Assessment (https://oehha.ca.gov/calenviroscreen/report/calenviroscreen-30). Block groups are nested within census tracts, larger geographic units that contain between 1,200 and 8,000 people. We assigned block groups the CalEnviroScreen score of their census tract in order to compare CalEnviroScreen rankings near GHG facilities to those of neighborhoods in the rest of the state.

Some oil and gas facilities report GHG emissions on an aggregate basis. In order to more accurately characterize neighborhood demographics near the sites of the pollutant emissions, we obtained information on the geographic location of drilling sites for 19 oil and gas facilities from CARB. These drilling site locations were included when ascertaining whether a block group was near a regulated facility. If a block group contained or was near several drilling sites belonging to 1 facility, we considered it to be near 1 GHG facility rather than multiple.

### Allowances and offsets

Information on the allocation of allowances was compiled from the California Code of Regulations (17 CA ADC § 95841 and 17 CCR § 95870) and CARB publications on the public allocation of allowances and estimates of state-owned allowances [26–28]. We obtained the number of allowances and offsets surrendered by each company at the completion of the first compliance period from CARB’s 2013–2014 compliance report [29]. Information on individual offset projects was compiled from CARB documents on offsets issued as of August 10, 2016 [30], and individual project descriptions provided in the American Carbon Registry and Climate Action Reserve carbon offset registries (http://americancarbonregistry.org; http://www.climateactionreserve.org).

### Analysis

The construction of the final dataset for analysis is shown in S1 Fig. All statistical analyses were conducted in R (R Foundation; https://www.r-project.org). Mann–Whitney–Wilcoxon tests were used to test for differences in neighborhood demographics near facilities because demographic variables were not normally distributed. Emissions data were also highly skewed, so we used log values in our analysis in order to reduce the non-normality of model residuals. For the cross-sectional analysis, a series of simple linear regressions was used to examine the correlation between GHG and co-pollutant emissions cross-sectionally for each industry category, with log(t co-pollutant) as the outcome and log(t GHG) as the predictor variable using the most recent year with both values available and greater than 0 for each facility.

For the longitudinal analysis, a series of mixed effects regression models was used to estimate the correlation between GHG and co-pollutant emissions over time, with log(t co-pollutant) as the outcome and log(t GHG) as the predictor variable. We included a random slope and a random intercept for facility. This approach allowed us to incorporate missing values during years when facilities did not report; missing values were assumed to be missing at random. In order to be able to incorporate 0 values, a small constant equivalent to the reporting threshold divided by the square root of 2 was substituted for 0 values following convention for left-censored, skewed environmental data. The reporting threshold was considered to be 0.05 in the original units of each data source (short tons for CEIDARs, metric tons for GHGs, and pounds for air toxics) since the lowest reported values were 1, and we assumed values below 0.5 would have been rounded down to 0. We conducted a sensitivity analysis to examine the effect of the imputed value for 0s on our results. Coefficients from the regression models can be interpreted as estimates of the percent change in co-emissions associated with a 1% change in emitter covered GHG emissions, either comparing across facilities in the case of the cross-sectional analysis or over time in the case of the longitudinal analysis.

Finally, we also applied a multivariable logistic modeling strategy to assess the independent effect of multiple block group demographic variables on the odds of an increase in annual average GHGs and co-pollutant emissions from nearby facilities before (January 1, 2011–December 31, 2012) versus after (January 1, 2013–December 31, 2015) implementation of the cap-and-trade program.

## Results

### Facilities regulated under California’s cap-and-trade program are disproportionately located in disadvantaged communities

**Fig 1:**
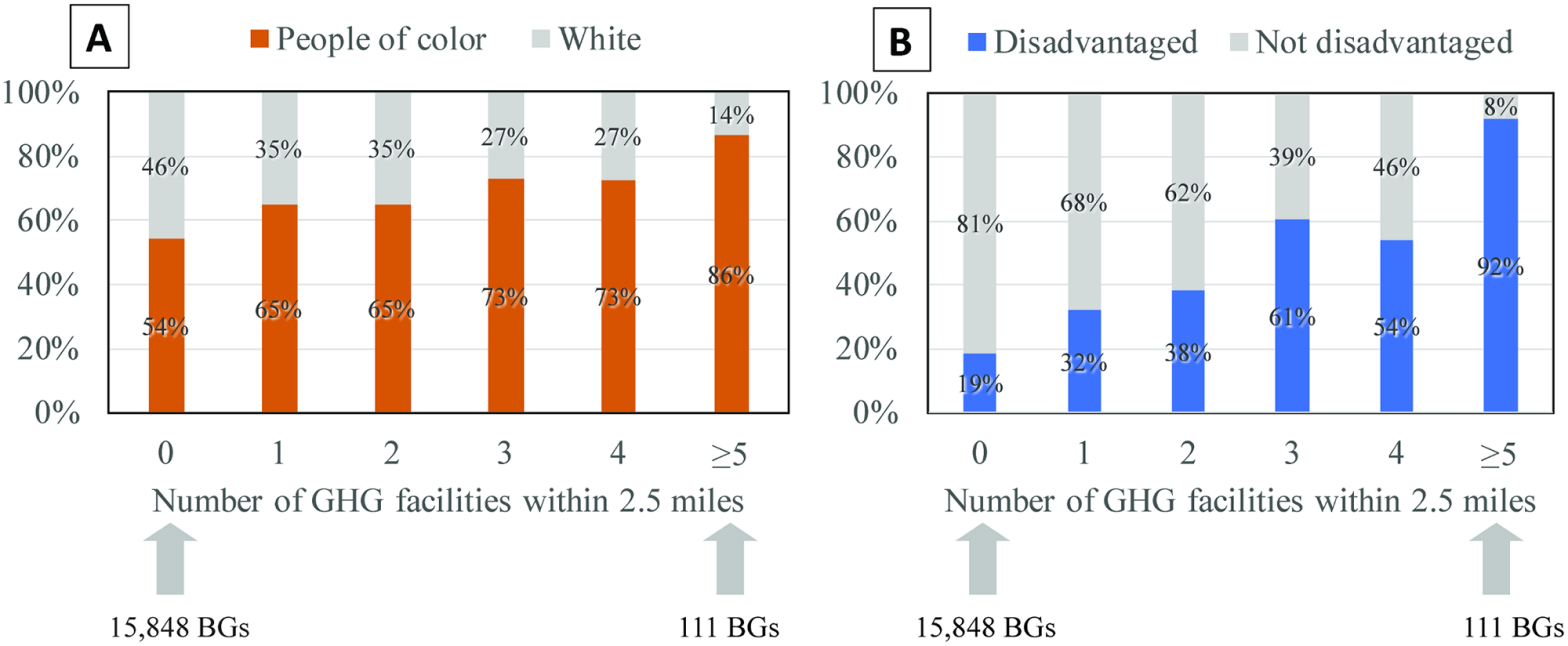
Disadvantaged neighborhoods host a disproportionate number of facilities covered by California’s cap-and-trade program. Facilities were assigned to neighborhoods (census block groups) if they were located within them or within 2.5 miles of the geographic block group centroid of nearby block groups. Neighborhoods were compared based on their (A) racial/ethnic composition using American Community Survey 2011–2015 5-year estimates and (B) community disadvantage (includes block groups within the highest scoring 25% of census tracts based on CalEnviroScreen 3.0 score [31]) (*n* = 322 facilities; 38,066,920 residents; 23,190 US Census block groups). BG, census block group; GHG, greenhouse gas.

GHG-emitting facilities regulated under California’s cap-and-trade program are disproportionately located in disadvantaged communities (Fig 1). The relative differences between neighborhoods within 2.5 miles (4.0 km) (based on their geographic census block group centroids) of a regulated facility as compared to neighborhoods located beyond 2.5 miles were, on average, 59% higher in population density, 34% higher in the proportion of residents of color, 23% higher in the proportion of poor residents, 64% higher in the proportion of residents with low educational attainment, and 80% higher in the proportion of linguistically isolated households in which no one age 14 years or older speaks English very well (Table 1). A higher proportion of neighborhoods near facilities were designated by CalEnviroScreen as disadvantaged (38% versus 19% of neighborhoods not near facilities) (Table 1). Similar but generally smaller relative differences also existed at the smaller, 1-mile (1.6 km), buffer distance; one exception was a larger relative difference in the proportion of poor residents within 1 mile (54%) (see S1 Table).

**Table 1:** Characteristics of neighborhoods (US Census block groups) near facilities regulated by California’s cap-and-trade program, 2011–2015.

Characteristic	Within 2.5 miles of a facility^a^ (*n* = 7,342 BGs; *n* = 11,765,168 people)	Beyond 2.5 miles of a facility^a^ (*n* = 15,848 BGs; *n* = 26,652,068 people)	*p*-Value^b^
Median (IQR) population density (people/km^2^)	3,627 (2,118–6,423)	2,285 (901–3,890)	<0.001
Median (IQR) percent people of color	71 (44–91)	53 (30–80)	<0.001
Median (IQR) percent poor^c^	37 (19–58)	30 (16–49)	<0.001
Median (IQR) percent low educational attainment^d^	18 (6–36)	11 (5–25)	<0.001
Median (IQR) percent linguistically isolated households^e^	9 (3–18)	5 (1–13)	<0.001
Percent BGs designated as a disadvantaged community^f^	38	19	<0.001^g^

^a^Based on their geographic census block group centroids. Block groups are generally contiguous geographic areas that contain between 600 and 3,000 people and can vary in size depending on population density. Neighborhood characteristics were obtained from the American Community Survey 2011–2015 5-year estimates.

^b^Two-tailed Mann–Whitney–Wilcoxon test.

^c^Percent of residents living below twice the federal poverty level.

^d^Percent of residents older than 25 years without a high school education.

^e^Percent of population living in households where no one age ≥14 years speaks English very well.

^f^Based on CalEnviroScreen 3.0 [19].

^g^Pearson’s chi-squared test with Yates’s continuity correction.

BG, census block group; IQR, interquartile range.

### Most regulated facilities increased their local GHG emissions

**Fig 2:**
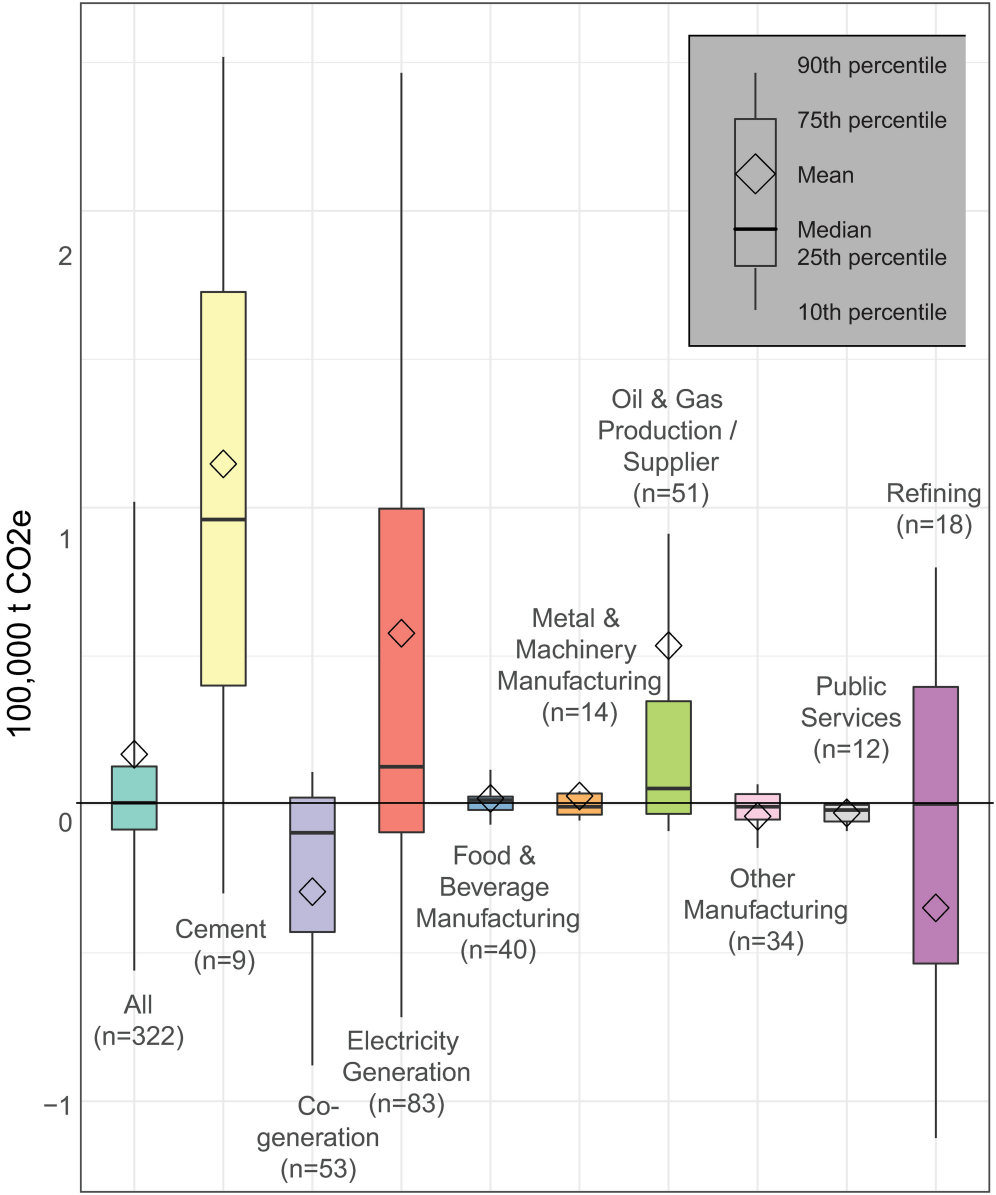
Changes in annual average greenhouse gas emissions within California after implementation of the state’s cap-and-trade program. Facility emissions 3 years after carbon trading began (2013–2015) are compared to those from the 2 years prior to the initiation of trading (2011–2012). Due to differences in accounting, comparable emission estimates are not available prior to 2011. *n* = number of facilities in each industry sector. t CO_2e_, metric tons CO_2_ equivalent.

The majority of facilities (52%) had higher annual average local GHG emissions (a change in mean aggregate emissions of 6,773,670 t) after implementation (2013–2015) of the cap-and-trade program as compared to the 2 years prior to implementation (2011–2012) (S4 Table). A majority of facilities also increased their annual average PM_2.5_, VOC, and air toxics emissions during this time period (51%, 57%, and 52%, respectively), while a minority increased their annual average NO_x_ and SO_x_ emissions (46% and 44%, respectively). While the program has claimed an overall reduction in total annual average GHG emissions during this period, this decrease was primarily achieved through indirect reductions associated with cutbacks in purchases of more carbon intensive electricity (such as electricity generated from coal-fired rather than natural gas power plants) imported from outside the state, rather than reductions in local emissions within California (see S2 Fig). Changes in GHG emissions within California during this period varied by industry sector. For example, while 70% of co-generation facilities decreased annual average emissions in 2013–2015 relative to 2011–2012, 75% of cement plants increased emissions. Cement plants had the highest median increase in local GHG emissions, followed by electricity generators, oil and gas producers, food and beverage manufacturing, and refineries (Fig 2).

### GHG and co-pollutant emissions are correlated

GHGs and hazardous co-pollutants emitted by facilities regulated under California’s cap-and-trade program were positively correlated when comparing across facilities. The strength of the correlation between GHG and co-pollutant emissions across facilities varied by co-pollutant and industry sector. Co-pollutant emissions tended to rise most steeply with GHG emissions among public service facilities (for PM_2.5_), metal and machinery manufacturing facilities (for NO_x_), refineries (for SO_x_), co-generation facilities (for VOCs), and other manufacturing facilities (for air toxics), while co-pollutant emissions overall tended to be the most tightly correlated with GHG emissions (based on the model *R*^2^) among cement plants and refineries (Table 2).

**Table 2:** Correlation between local greenhouse gas (metric tons CO_2_ equivalent) and co-pollutant (metric tons) emissions in 2015 from facilities regulated under California’s cap-and-trade program.

Industry (*n*)^a^	PM_2.5_		NO_x_		SO_x_		VOCs		Air toxics^b^	
	β (95% CI)	*R*^2^	β (95% CI)	*R*^2^	β (95% CI)	*R*^2^	β (95% CI)	*R*^2^	β (95% CI)	*R*^2^
Cement plants (9)	0.43 (0.22, 0.64)	0.65	0.86 (0.81, 0.91)	0.99	1.36 (−0.63, 3.36)	0.10	0.61 (0.38, 0.83)	0.77	1.18 (0.89, 1.47)	0.90
Co-generation (53)	0.51 (0.17, 0.84)	0.13	0.39 (0.05, 0.74)	0.07	0.76 (0.42, 1.09)	0.27	0.71 (0.27, 1.14)	0.17	−0.01 (−4.19, 4.17)	−0.25
Electricity generation (83)	0.50 (0.36, 0.64)	0.36	0.52 (0.38, 0.66)	0.38	0.48 (0.33, 0.62)	0.35	0.48 (0.33, 0.62)	0.33	—^c^	—^c^
Food and beverage manufacturing (40)	0.80 (0.49, 1.12)	0.38	0.30 (−0.23, 0.84)	0.01	0.78 (0.21, 1.34)	0.14	0.36 (−0.42, 1.14)	0.00	−0.11 (−3.53, 3.32)	−0.10
Metal and machinery manufacturing (14)	0.00 (−0.28, 0.28)	−0.08	1.03 (0.10, 1.96)	0.22	2.07 (−0.17, 4.30)	0.15	−0.05 (−0.28, 0.17)	−0.06	0.12 (−0.77, 1.00)	−0.12
Oil and gas production/suppliers (49)	0.75 (0.49, 1.01)	0.4	0.61 (0.29, 0.94)	0.21	0.89 (0.58, 1.21)	0.40	0.44 (0.11, 0.78)	0.11	—^c^	—^c^
Other manufacturing (34)	0.34 (0.02, 0.66)	0.1	0.82 (0.41, 1.24)	0.30	1.11 (0.17, 2.06)	0.13	−0.17 (−0.73, 0.38)	−0.02	2.07 (0.41, 3.73)	0.19
Public services (12)	1.08 (0.26, 1.09)	0.34	0.53 (−0.39, 1.46)	0.02	0.98 (−0.03, 1.99)	0.19	1.14 (−0.08, 2.36)	0.17	—^c^	—^c^
Refineries (18)	0.84 (0.68, 1.00)	0.87	0.64 (0.53, 0.74)	0.89	1.15 (0.85, 1.45)	0.77	0.39 (0.25, 0.52)	0.65	1.09 (0.41, 1.76)	0.37

We show log-linear regression coefficients (β), 95% confidence intervals, and model fit (*R*^2^). For facilities that were missing data for 2015, we used data from the most recent year with non-0 emission values for both sets of pollutants. Eight facilities categorized as hydrogen plants or other are not shown because there were fewer than 5 facilities with data in either industry category.

^a^*n* refers to the highest total number of facilities in that category. For some pollutants, the *n* may be smaller due to missing data. Air toxics are only reported by a small proportion of facilities, and the *n*’s were as follows: cement plants (8), co-generation (6), electricity generation (4), food and beverage manufacturing (12), metal and machinery manufacturing (10), oil and gas production/suppliers (2), other manufacturing (22), public services (0), and refineries (16).

^b^In all, 595 individual chemicals and 32 chemical categories known to cause cancer, other acute or chronic human health effects, and/or significant adverse environmental effects are included as air toxics and reported under the federal Toxics Release Inventory by a subset of facilities [32]. Some air toxics are also VOCs, which are defined by the US Environmental Protection Agency as “any compound of carbon, excluding carbon monoxide, carbon dioxide, carbonic acid, metallic carbides or carbonates, and ammonium carbonate, which participates in atmospheric photochemical reactions” [33].

^c^Estimate not reported because there were fewer than 5 facilities with pollutant emission data.

PM2.5, particulate matter <2.5 micrometers; NO_x_, nitrogen oxides; SO_x_, sulfur oxides; VOC, volatile organic compound.

**Fig 3:**
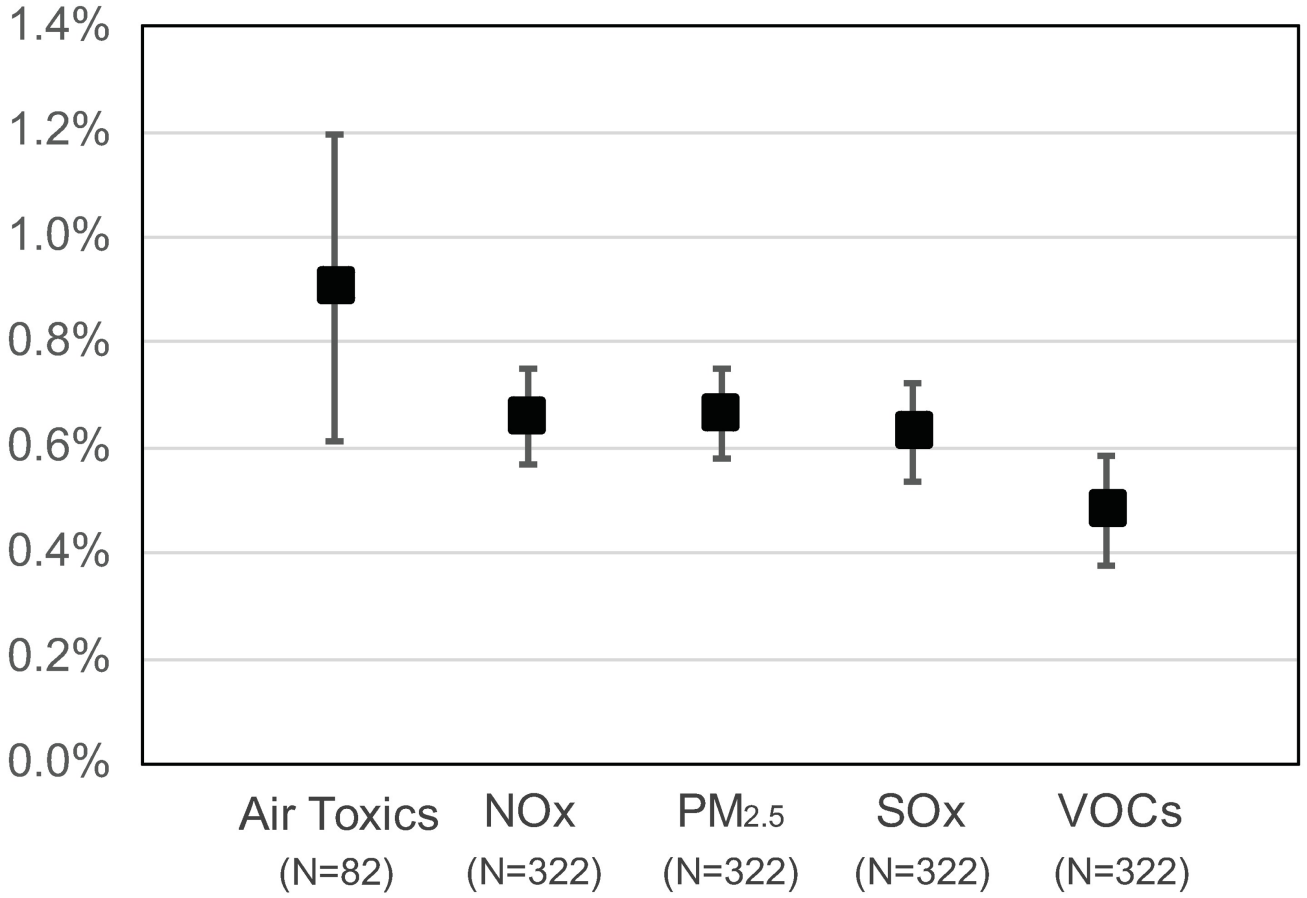
Mean percent change and 95% confidence interval of co-pollutant emissions per 1% change in greenhouse gas emissions. Estimates were obtained from a mixed effects regression model of annual panel (longitudinal) data from 2011–2015 with a random intercept and random slope for facility. *N*’s for each pollutant category refer to the number of facilities. PM2.5, particulate matter <2.5 micrometers; NO_x_, nitrogen oxides; SO_x_, sulfur oxides; VOC, volatile organic compound.

GHG and co-pollutant emissions were also correlated within facilities over time. On average, a 1% change in annual GHG emissions at the facility level was accompanied by a 0.91%, 0.66%, 0.66%, 0.63%, and 0.48% change in air toxics, NO_x_, PM_2.5_, SO_x_, and VOCs, respectively (all *p* < 0.001) (see Fig 3). This association indicates that reductions in GHG emissions can be expected to result in reductions in co-pollutant emissions (and vice versa). Imputing slightly higher values for reported emissions of 0 in the analysis did not affect these effect estimates by more than 3%. We did not observe a one-to-one relationship between changes in GHG and co-pollutant emissions, which may be a result of the fact that emission reductions can be achieved using diverse strategies. For example, a facility may use scrubbers to reduce PM emissions, but potentially increase GHG emissions in the process because this pollution reduction strategy is more energy intensive. Alternatively, a facility could undertake energy conservation efforts that reduce energy use and thereby GHG emissions, but have little impact on air toxics or other co-pollutant emissions that result from processes unrelated to energy production.

### Emission patterns differ by neighborhood demographics

Given the spatially clustered nature of facilities regulated under California’s cap-and-trade program, we examined the relationship between neighborhood demographics and changes in emissions from all facilities located nearby (≤2.5 miles from the geographic block group centroid). We found that compared to neighborhoods that experienced decreases in aggregate annual average GHG emissions after versus before implementation of carbon trading, neighborhoods that experienced increases of both annual average GHGs and annual average co-pollutants were more likely to be designated by CalEnviroScreen as disadvantaged and had higher proportions of residents of color, higher rates of poverty, higher rates of low educational attainment, and higher proportions of linguistically isolated households (Table 3). Population density was generally lower in the neighborhoods that experienced emission increases, except for those where both GHGs and air toxics from nearby facilities increased. Similar differences were also noted using a 1-mile buffer distance (see S2 Table). Logistic modeling results assessing the odds of block groups experiencing increases of GHGs and each co-pollutant compared to block groups that experienced decreases in either indicate that less densely populated block groups and those with higher proportions of residents of color and residents with low levels of educational attainment had an increased odds of experiencing an increase in GHGs and co-pollutants independent of the proportion of poor or linguistically isolated residents (Table 4). Results for other demographic variables such as the proportion of residents living in poverty and the proportion of linguistically isolated households were less consistent across models for each of the co-pollutants. Overall, among those block groups that experienced increases in GHG and co-pollutant emissions, the median and range of emission changes in metric tons was as follows: GHGs, 14,320 (1.7–1,137,000); PM_2.5_, 2.78 (0.03–113.6); NO_x_, 5.35 (0.03–223.2); SO_x_, 0.51 (<0.001–132.7); VOCs, 2.96 (0.015–296); and air toxics, 0.37 (0.001–95.6).

**Table 3:** Characteristics of neighborhoods (US Census block groups) that experienced an increase in annual average aggregate emissions from regulated facilities within 2.5 miles after (2013–2015) as compared to before (2011–2012) implementation of carbon trading.

Characteristic	GHGs decreased (reference group) (*n* = 3,992 BGs; 6,288,141 people)	GHGs increased (*n* = 2,389 BGs; 4,024,069 people)	GHGs and PM_2.5_ increased (*n* = 1,360 BGs; 2,345,505 people)	GHGs and NO_x_ increased (*n* = 1,499 BGs; 2,519,957 people)	GHGs and SO_x_ increased (*n* = 1,219 BGs; 2,121,769 people)	GHGs and VOCs increased (*n* = 1,708 BGs; 2,881,139 people)	GHGs and air toxics increased (*n* = 581 BGs; 964,916 people)
Median (IQR) population density (people/km^2^)	4,014 (2,340–6,857)	3,376*** (1,966–5,715)	3,368*** (1,966–5,900)	3,342*** (2,035–5,337)	2,794*** (1,701–4,368)	3,668*** (2,125–6,385)	4,371 (2,397–7,553)
Median (IQR) percent people of color	68 (42–90)	79*** (50–95)	85*** (59–96)	78*** (52–93)	76*** (56–91)	85*** (60–96)	92*** (71–98)
Median (IQR) percent poor	36 (18–57)	42*** (20–63)	45*** (25–66)	40*** (21–61)	44*** (23–65)	43*** (22–65)	47*** (24–64)
Median (IQR) percent low education	16 (5–34)	23*** (8–43)	27*** (11–47)	21*** (8–40)	25*** (−9–40)	25*** (10–47)	32*** (15–52)
Median (IQR) percent linguistically isolated	8 (3–18)	11*** (−4–21)	12*** (4–21)	10 (4–19)	10*** (4–18)	12*** (5–22)	13*** (5–23)
Percent BGs designated as a disadvantaged community	31	47***	54***	42***	42***	49***	66***

We exclude 19 oil and gas facilities with multiple sub-facility locations because they report their GHG emissions on an aggregated basis such that emission changes cannot be determined in the neighborhoods near the sub-facility locations where they occur.

***p < 0.001 compared to block groups where GHGs decreased, from Pearson’s chi-squared test with Yates’s continuity correction (for disadvantaged communities) or 2-tailed Mann–Whitney–Wilcoxon test (all other variables).

BG, census block group; GHG, greenhouse gas; IQR, interquartile range; NO_x_, nitrogen oxides; PM2.5, particulate matter <2.5 micrometers; SO_x_, sulfur oxides; VOC, volatile organic compound.

**Table 4:** Neighborhood (block group) demographic predictors of increases in GHG and co-pollutant emissions from facilities within 2.5 miles obtained from multivariable logistic regression.

Predictor	GHGs and PM_2.5_ increased	GHGs and NO_x_ increased	GHGs and SO_x_ increased	GHGs and VOCs increased	GHGs and air toxics increased
(Intercept)	0.24 (0.23, 0.26)	0.30 (0.28, 0.31)	0.20 (0.18, 0.21)	0.34 (0.32, 0.36)	0.08 (0.07, 0.09)
Population density	0.65 (0.59, 0.72)	0.70 (0.64, 0.76)	0.38 (0.34, 0.43)	0.77 (0.71, 0.83)	0.98 (0.88, 1.08)
% people of color	1.19 (1.08, 1.31)	1.13 (1.04, 1.24)	1.12 1.01, 1.23)	1.38 (1.26, 1.51)	1.78 (1.53, 2.08)
% poor	0.90 (0.81, 0.99)	1.02 (0.93, 1.12)	1.37 (1.24, 1.51)	0.85 (0.78, 0.93)	0.60 (0.51, 0.69)
% low education	1.75 (1.55, 1.97)	1.22 (1.09, 1.36)	1.09 (0.96, 1.23)	1.40 (1.25, 1.56)	1.94 (1.64, 2.29)
% linguistically isolated	0.87 (0.80, 0.95)	0.89 (0.82, 0.97)	0.91 (0.83, 1.00)	1.00 (0.92, 1.08)	0.82 (0.73, 0.92)

All demographic variables are scaled. Effect estimates are given as odds ratio (95% CI) and can be interpreted as the odds associated with a 1-standard-deviation increase in each predictor. The reference group for each model is block groups that experienced decreases in either GHGs or the co-pollutant. *n* = 303 facilities for all models except air toxics, which includes 82 facilities that are required to report emissions to the Toxics Release Inventory. We exclude 19 oil and gas facilities with multiple sub-facility locations because they report their GHG emissions on an aggregated basis such that emission changes cannot be determined in the neighborhoods near the sub-facility locations where they occur.

GHG, greenhouse gas; NO_x_, nitrogen oxides; PM2.5, particulate matter <2.5 micrometers; SO_x_, sulfur oxides; VOC, volatile organic compound.

### Offsets can undercut emission reduction efforts and environmental equity goals

During the first compliance period (2013–2014), offset credits represented more than 4.4% of the total compliance obligation (credits and offsets surrendered for each metric ton of GHGs emitted), or over 4 times the targeted reduction as established by the cap in GHG emissions from 2013 to 2014. The majority of the offset credits (75.6%) were generated by out-of-state projects. Overall, most offset projects were in forestry (46.3%) and destruction of ozone-depleting substances (45.6%). Facilities owned by companies that used offsets emitted significantly higher levels of GHGs than those owned by companies that did not use offsets (see S3 Table). For example, the 10 companies using the most offset credits during the first compliance period were responsible for 82% of offsets surrendered and 43% of total covered GHG emissions. Facilities owned by companies that used offset credits also emitted more PM_2.5_, NO_x_, SO_x_, and air toxics over the same time period, although these differences were not statistically significant. Conversely, average VOC emissions were lower among companies that used offsets (*p* = 0.001). While companies using offsets tended to be larger emitters overall, their annual average changes in GHGs and co-pollutant emissions after (2013–2015) versus before (2011–2012) the implementation of carbon trading were statistically indistinguishable from those of companies not using offsets (data not shown).

## Discussion

California’s efforts to slow climate change by reducing GHG emissions have the potential to bring about significant air quality and health benefits to the state’s less advantaged residents. GHG-emitting facilities tend to be located in neighborhoods with higher proportions of residents living in poverty and people of color, and the temporal correlation between GHG and co-pollutant emissions indicates that incentivizing deeper reductions in local GHG emissions could bolster the environmental equity goals articulated in California’s climate change laws. Our results, however, indicate that, thus far, the cap-and-trade program has not yielded this set of localized improvements in environmental equity.

Prior analyses of emission trading programs found little evidence that they produced socially inequitable outcomes. For example, studies of the US Acid Rain Program to reduce sulfur dioxide emissions from coal-fired power plants and of Southern California’s Regional Clean Air Incentives Market (RECLAIM) program to reduce NO_x_ and SO_x_ emissions from large facilities such as power plants, refineries, and manufacturing facilities found no evidence that the locations of emissions or purchases of allowances were disparate with respect to the racial/ethnic makeup or income of surrounding neighborhoods [34–36]. One exception is an analysis that incorporated dispersion modelling of emissions and found that high-income neighborhoods benefitted more from RECLAIM than did low-income neighborhoods and that, conditional on income, African American individuals benefitted more, and Hispanic individuals benefitted less, than white individuals [37]. Our analytical approach differs from that taken in most prior studies because we use a neighborhood—rather than facility-level—perspective to evaluate changes in aggregate emissions. Such an approach is warranted in our context because polluting facilities are clustered in space and many Californians live in close proximity to multiple facilities, as shown in Fig 1.

Our results also suggest that although California’s total GHG emissions are below the cap set by the cap-and-trade program, results have been underwhelming with respect to local (in-state) GHG emissions, which increased on average for regulated facilities in several industry sectors (with a net increase of mean local GHG emissions of 6.7 million t CO_2e_ from 2011–2012 to 2013–2015, and a median facility-level increase of 600 t across all facilities that we analyzed). The lack of deeper reductions in local emissions may be due to an initial overallocation of allowances that resulted in an oversupply of cheap allowances on the market. Emissions at the initiation of the carbon trading program were lower than expected due to the economic downturn related to the Great Recession of 2008, and the initial allocation of allowances was thus far greater than the metric tons of regulated emissions. There was a larger aggregate decrease in local GHG emissions in 2015 compared to prior years (see S2 Fig), suggesting that greater reductions may be achieved going forward as the cap is lowered further. However, banking of excess allowances from early years of the program [38] and the substantial use of offset credits suggest that there may continue to be little reduction in in-state emissions. The quantity of offsets allowed thus far under the program is worrisome because the validity of GHG emission reductions claimed under offset projects is controversial given the challenge of verifying if they are truly additional and would not have occurred in the absence of the cap-and-trade program [39–42]. Offset credits included in our analysis were primarily generated from forestry projects outside the state that do not offer the same benefits as localized co-pollutant emission reductions. Recent California legislation (AB 398) seeks to address this issue by reducing the use of offset credits generally, while also increasing the proportion of allowable offsets that are generated from in-state projects [43]. Under the current cap-and-trade program, offset credits can make up as much as 8% of the total amount of allowances used for compliance by a regulated company. However, AB 398 will reduce this amount. From 2021 to 2025, up to 4% of a covered company’s compliance obligation can be met by offsets, and half of these must be in state or “provide direct environmental benefits” to California. From 2026 to 2030, up to 6% of a covered company’s compliance obligations can be met by offsets, with at least half generated from in-state projects.

In summary, our study results reflect preliminary local GHG and co-pollutant emissions and social equity patterns of the first 3 years of California’s cap-and-trade program for which data are currently available. One limitation of our analysis is that it was restricted to regulated industries and was not able to include an assessment of the emission patterns and equity implications of GHG reductions from transportation-related sources. In addition, ongoing investments of a significant portion of California’s cap-and-trade revenue in disadvantaged communities as mandated by law [18] to mitigate climate change could also potentially incentivize deeper local GHG and co-pollutant reductions in the future. As data to examine these issues become available, future research can more holistically assess the extent to which GHG and co-pollutant emission patterns from both industrial and transportation sources may be shifting due to changes in industrial production decisions, cap-and-trade revenue investments, and policy initiatives that encourage deeper in-state emission reductions, particularly in disadvantaged communities.

Some analysts have cautioned against integrating air quality into climate policy, and argue that co-pollutants are best regulated under existing laws such as the US Clean Air Act [44]. However, others note that the most cost-effective climate regulation would achieve GHG reductions in locations where the health benefits are greatest [15]. Our analysis suggests that California’s climate policy could better harmonize efforts to reduce GHGs with improvements to local air quality, and that market-based strategies in general could provide greater overall benefits by incentivizing localized GHG reductions in disadvantaged and highly polluted neighborhoods. For example, other emission trading programs have restricted trading and raised the price of allowances within high-pollution areas in order to promote deeper reductions in disproportionately impacted neighborhoods [45]. In addition, regulated firms could be required or incentivized to purchase offsets that are linked to local projects that reduce GHG emissions and also improve air quality in the regions where their facilities are located; such local offset projects could include electrification of railyards and ports, cleaning up truck fleets, or financing retrofits to reduce GHGs and co-pollutant emissions from other local emission sources. Such local offset projects could enhance government oversight and promote community partnerships in project monitoring and emission verification.

The administrative costs of such an integrated strategy are likely to be modest, particularly since a small number of industry sectors and facilities present the greatest opportunities to achieve air quality co-benefits [15]. It would require more systematic temporal and spatial tracking of the air quality and environmental equity impacts of cap-and-trade through annual and verifiable GHG and co-pollutant emission reporting by each regulated facility, combined with facility- and company-specific allowance allocations and trading information, including the use of offsets. These data are beginning to be made publicly available, which will enable more effective and timely regulatory oversight of emission temporal patterns and future research on the health and environmental equity impacts of cap-and-trade. Ultimately, applying regulatory and analytical tools that address the contributions of GHG emission sources to local cumulative air pollution burdens could support better integration of the sustainability and environmental equity goals of California’s climate laws and inform carbon pricing efforts elsewhere.

## Supporting information

S1 Data Emission data used in this analysis. (XLSX)

S2 Data Oil and gas facility locations. (XLSX)

S1 Fig Construction of the dataset. (PDF)

S2 Fig GHG allowances and emissions covered under California’s cap-and-trade program, 2011–2015. (PDF)

S1 Table Characteristics of block groups ≤1 mile from facilities regulated by California’s cap-and-trade program. (PDF)

S2 Table Characteristics of block groups that experienced an increase in annual average aggregate emissions from regulated facilities within 1 mile after (2013–2015) as compared to before (2011–2012) implementation of carbon trading. (PDF)

S3 Table Differences in annual air pollutant emissions from facilities regulated under California’s cap-and-trade program by offset usage, 2013–2014. (PDF)

S4 Table Changes in mean aggregate air pollutant emissions from all regulated facilities after the implementation of California’s cap-and-trade program in 2013. (PDF)

## Abbreviations

BG: Census block group
CARB: California Air Resources Board
CEIDARS: California Emission Inventory Development and Reporting System
CO_2e_: CO_2_ equivalent
GHG: greenhouse gas
IQR: interquartile range
MRR: mandatory reporting regulation
NO_x_: nitrogen oxide(s)
PM: particulate matter
RSEI: Risk-Screening Environmental Indicators
SO_x_: sulfur oxide(s)
t: metric ton
VOC: volatile organic compound

## References

[1] Smith KR, Frumkin H, Balakrishnan K, Butler CD, Chafe ZA, Fairlie I, et al Energy and human health. Annu Rev Public Health. 2013;34:159–88. doi: 10.1146/annurev-publhealth-031912-114404 23330697 10.1146/annurev-publhealth-031912-114404

[2] Smith KR, Haigler E. Co-benefits of climate mitigation and health protection in energy systems: scoping methods. Annu Rev Public Health. 2008;29:11–25. doi: 10.1146/annurev.publhealth.29.020907.090759 18173381 10.1146/annurev.publhealth.29.020907.090759

[3] Nemet GF, Holloway T, Meier P. Implications of incorporating air-quality co-benefits into climate change policymaking. Environ Res Lett. 2010;5:014007 doi: 10.1088/1748-9326/5/1/014007

[4] Zapata C, Muller N, Kleeman MJ. PM2.5 co-benefits of climate change legislation part 1: California’s AB 32. Clim Change. 2013;117:377–97. doi: 10.1007/s10584-012-0545-y

[5] Burtraw D, Linn J, Palmer K, Paul A. The costs and consequences of Clean Air Act regulation of CO2 from power plants. Am Econ Rev. 2014;104:557–62. doi: 10.1257/aer.104.5.557

[6] Hajat A, Hsia C, O’Neill MS. Socioeconomic disparities and air pollution exposure: a global review. Curr Environ Health Rep. 2015;2:440–50. doi: 10.1007/s40572-015-0069-5 26381684 10.1007/s40572-015-0069-5 PMC4626327

[7] Deguen S, Zmirou-Navier D. Social inequalities resulting from health risks related to ambient air quality—a European review. Eur J Public Health. 2010;20:27–35. doi: 10.1093/eurpub/ckp220 20081212 10.1093/eurpub/ckp220

[8] US Environmental Protection Agency. Our nation’s air: status and trends through 2010. Washington (DC): US Environmental Protection Agency; 2012 Feb [cited 2018 Jun 12]. Report No. EPA-454/R-12-001. https://nepis.epa.gov/Exe/ZyPURL.cgi?Dockey=P100E174.TXT.

[9] American Lung Association. State of the air 2017. Chicago: American Lung Association; 2017 [cited 2018 Jun 12]. 164 p. http://www.lung.org/assets/documents/healthy-air/state-of-the-air/state-of-the-air-2017.pdf.

[10] Grineski SE, Collins TW, Morales DX. Asian Americans and disproportionate exposure to carcinogenic hazardous air pollutants: a national study. Soc Sci Med. 2017;185:71–80. doi: 10.1016/j.socscimed.2017.05.042 28554161 10.1016/j.socscimed.2017.05.042 PMC5523857

[11] Clark LP, Millet DB, Marshall JD. National patterns in environmental injustice and inequality: outdoor NO2 air pollution in the United States. PLoS ONE. 2014;9:e94431 doi: 10.1371/journal.pone.0094431 24736569 10.1371/journal.pone.0094431 PMC3988057

[12] Levy JI, Wilson AM, Zwack LM. Quantifying the efficiency and equity implications of power plant air pollution control strategies in the United States. Environ Health Perspect. 2007;115:743–50. doi: 10.1289/ehp.9712 17520062 10.1289/ehp.9712 PMC1867973

[13] Levy JI, Greco SL, Melly SJ, Mukhi N. Evaluating efficiency-equality tradeoffs for mobile source control strategies in an urban area. Risk Anal. 2009;29:34–47. doi: 10.1111/j.1539-6924.2008.01119.x 18793281 10.1111/j.1539-6924.2008.01119.x PMC3690594

[14] Stavins RN. A meaningful U.S. cap-and-trade system to address climate change. Rochester (NY): Social Science Research Network; 2008 10 [cited 2018 Jun 12]. Report No. 1281518. https://papers.ssrn.com/abstract=1281518.

[15] Boyce JK, Pastor M. Clearing the air: incorporating air quality and environmental justice into climate policy. Clim Change. 2013;120:801–14. doi: 10.1007/s10584-013-0832-2

[16] Shonkoff S, Morello-Frosch R, Pastor M, Sadd J. environmental health and equity implications of climate change and mitigation policies in California: a review of the literature. Clim Change. 2011;109:S485–503.

[17] Farber D. Pollution markets and social equity: analyzing the fairness of cap and trade. Ecol Law Q. 2012;1:48–53.

[18] California Environmental Protection Agency. California climate investments to benefit disadvantaged communities. Sacramento: California Environmental Protection Agency; 2017 [cited 2017 Dec 11]. http://calepa.ca.gov/EnvJustice/GHGInvest/.

[19] California Environmental Protection Agency, Office of Environmental Health Hazard Assessment. CalEnviroScreen 3.0: update to the California Communities Environmental Health Screening Tool. Sacramento: Office of Environmental Health Hazard Assessment; 2017 1 [cited 2018 Jun 13]. 166 p. https://oehha.ca.gov/media/downloads/calenviroscreen/report/ces3report.pdf.

[20] Morello-Frosch R, Zuk M, Jerrett M, Shamasunder B, Kyle AD. Understanding the cumulative impacts of inequalities in environmental health: implications for policy. Health Aff (Millwood). 2011;30:879–87. doi: 10.1377/hlthaff.2011.0153 21555471 10.1377/hlthaff.2011.0153

[21] Woodruff TJ, Sutton P, Navigation Guide Work Group. An evidence-based medicine methodology to bridge the gap between clinical and environmental health sciences. Health Aff (Millwood). 2011;30:931–7. doi: 10.1377/hlthaff.2010.1219 21555477 10.1377/hlthaff.2010.1219 PMC6663095

[22] Vesterinen HM, Morello-Frosch R, Sen S, Zeise L, Woodruff TJ. Cumulative effects of prenatal-exposure to exogenous chemicals and psychosocial stress on fetal growth: systematic-review of the human and animal evidence. PLoS ONE. 2017;12:e0176331 doi: 10.1371/journal.pone.0176331 28700705 10.1371/journal.pone.0176331 PMC5507491

[23] California Air Resources Board. Mandatory greenhouse gas emissions reporting. Sacramento: California Air Resources Board; 2017 [cited 2017 Oct 25]. https://www.arb.ca.gov/cc/reporting/ghg-rep/ghg-rep.htm.

[24] 42 U.S.C. United States Code, 2011 edition. Chapter 116—Emergency Planning and Community Right-to-Know. Washington (DC): US Government Publishing Office; 2011 [cited 2018 Jun 13] https://www.govinfo.gov/content/pkg/USCODE-2011-title42/html/USCODE-2011-title42-chap116.htm.

[25] Padilla-Frausto ID, Wallace SP. The hidden poor: over three-quarters of a million older Californians overlooked by official poverty line. Policy Brief UCLA Cent Health Policy Res. 2015;(PB2015-03):1–8. 26376501

[26] California Air Resources Board. Public data on allowance allocation. Sacramento: California Air Resources Board; 2017 [cited 2017 Oct 25]. https://www.arb.ca.gov/cc/capandtrade/allowanceallocation/publicallocation.htm.

[27] California Air Resources Board. Estimated state auction budget. Sacramento: California Air Resources Board; 2017 [cited 2017 Oct 25]. https://www.arb.ca.gov/cc/capandtrade/stateauction.htm.

[28] California Air Resources Board. Annual allocation to electrical distribution utilities (EDU) under the cap-and-trade regulation. Sacramento: California Air Resources Board; 2015 [cited 2018 Jun 13]. https://www.arb.ca.gov/cc/capandtrade/allowanceallocation/edu-ng-allowancedistribution/electricity-allocation.pdf.

[29] California Air Resources Board. 2013–2014 compliance obligation detail for ARB’s cap-and-trade program. Sacramento: California Air Resources Board; 2018 [cited 2018 Jun 13]. https://www.arb.ca.gov/cc/capandtrade/2013-2014compliancereport.xlsx.

[30] California Air Resources Board. ARB offset credits issued. Sacramento: California Air Resources Board; 2016 [cited 2016 Aug 10]. https://www.arb.ca.gov/cc/capandtrade/offsets/issuance/arb_offset_credit_issuance_table.pdf.

[31] California Environmental Protection Agency. Designation of disadvantaged communities pursuant to Senate Bill 535 (De León). Sacramento: California Environmental Protection Agency; 2017 4 [cited 2018 Jun 13]. https://calepa.ca.gov/wp-content/uploads/sites/62/2017/04/SB-535-Designation-Final.pdf.

[32] US Environmental Protection Agency. Toxics Release Inventory (TRI) program: TRI-listed chemicals. Washington (DC): US Environmental Protection Agency; 2017 [cited 2017 Nov 10]. https://www.epa.gov/toxics-release-inventory-tri-program/tri-listed-chemicals.

[33] US Environmental Protection Agency. Indoor air quality (IAQ): technical overview of volatile organic compounds. Washington (DC): US Environmental Protection Agency; 2017 [cited 2017 Nov 10]. https://www.epa.gov/indoor-air-quality-iaq/technical-overview-volatile-organic-compounds.

[34] Corburn J. Emissions trading and environmental justice: distributive fairness and the USA’s acid rain programme. Environ Conserv. 2001;28:323–32. doi: 10.1017/S0376892901000352

[35] Ringquist EJ. Trading equity for efficiency in environmental protection? Environmental justice effects from the SO2 allowance trading program. Soc Sci Q. 2011;92:297–323. doi: 10.1111/j.1540-6237.2011.00769.x

[36] Fowlie M, Holland SP, Mansur ET. What do emissions markets deliver and to whom? Evidence from Southern California’s NOx trading program. Am Econ Rev. 2012;102:965–93. doi: 10.1257/aer.102.2.965

[37] Grainger C, Ruangmas T. Who wins from emissions trading? Evidence from California. Environ Resour Econ. 2017 9 1 doi: 10.1007/s10640-017-0180-1

[38] Busch C. Oversupply grows in the western climate initiative carbon market. San Francisco: Energy Innovation; 2017 [cited 2018 Jun 13]. 40 p. http://energyinnovation.org/wp-content/uploads/2017/12/Oversupply-Grows-In-The-WCI-Carbon-Market.pdf.

[39] Gillenwater M, Broekhoff D, Trexler M, Hyman J, Fowler R. Policing the voluntary carbon market. Nat Rep Clim Change. 2007;85–7. doi: 10.1038/climate.2007.58

[40] Zhang J, Wang C. Co-benefits and additionality of the clean development mechanism: an empirical analysis. J Environ Econ Manag. 2011;62:140–54. doi: 10.1016/j.jeem.2011.03.003

[41] Bento A, Kanbur R, Leard B. On the importance of baseline setting in carbon offsets markets. Clim Change. 2016;137:625–37. doi: 10.1007/s10584-016-1685-2

[42] Anderson CM, Field CB, Mach KJ. Forest offsets partner climate-change mitigation with conservation. Front Ecol Environ. 2017;15:359–65. doi: 10.1002/fee.1515

[43] California Assembly Bill No. 398 California Global Warming Solutions Act of 2006: market-based compliance mechanisms: fire prevention fees: sales and use tax manufacturing exemption. 2017 Jul 25 [cited 2018 Jun 13]. https://leginfo.legislature.ca.gov/faces/billNavClient.xhtml?bill_id=201720180AB398.

[44] Schatzki T, Stavins RN. Addressing environmental justice concerns in the design of California’s climate policy. Analysis Group; 2009 Oct [cited 2018 Jun 13]. 33 p. http://www.climatechange.ca.gov/eaac/comments/2009-11-03_Schatzki_and_Stavins_attachment.pdf.

[45] Gangadharan L. Analysis of prices in tradable emission markets: an empirical study of the regional clean air incentives market in Los Angeles. Appl Econ. 2004;36:1569–82. doi: 10.1080/0003684042000269466

