# Supplementary figures and images for: Carbon trading, co-pollutants, and environmental equity: Evidence from California’s cap-and-trade program (2011–2015)
Source: PLoS Med. 2018 Jul 10;15(7):e1002604. doi: 10.1371/journal.pmed.1002604 (PMC6038989; doi:10.1371/journal.pmed.1002604)

**Figure S1. Construction of the dataset.**

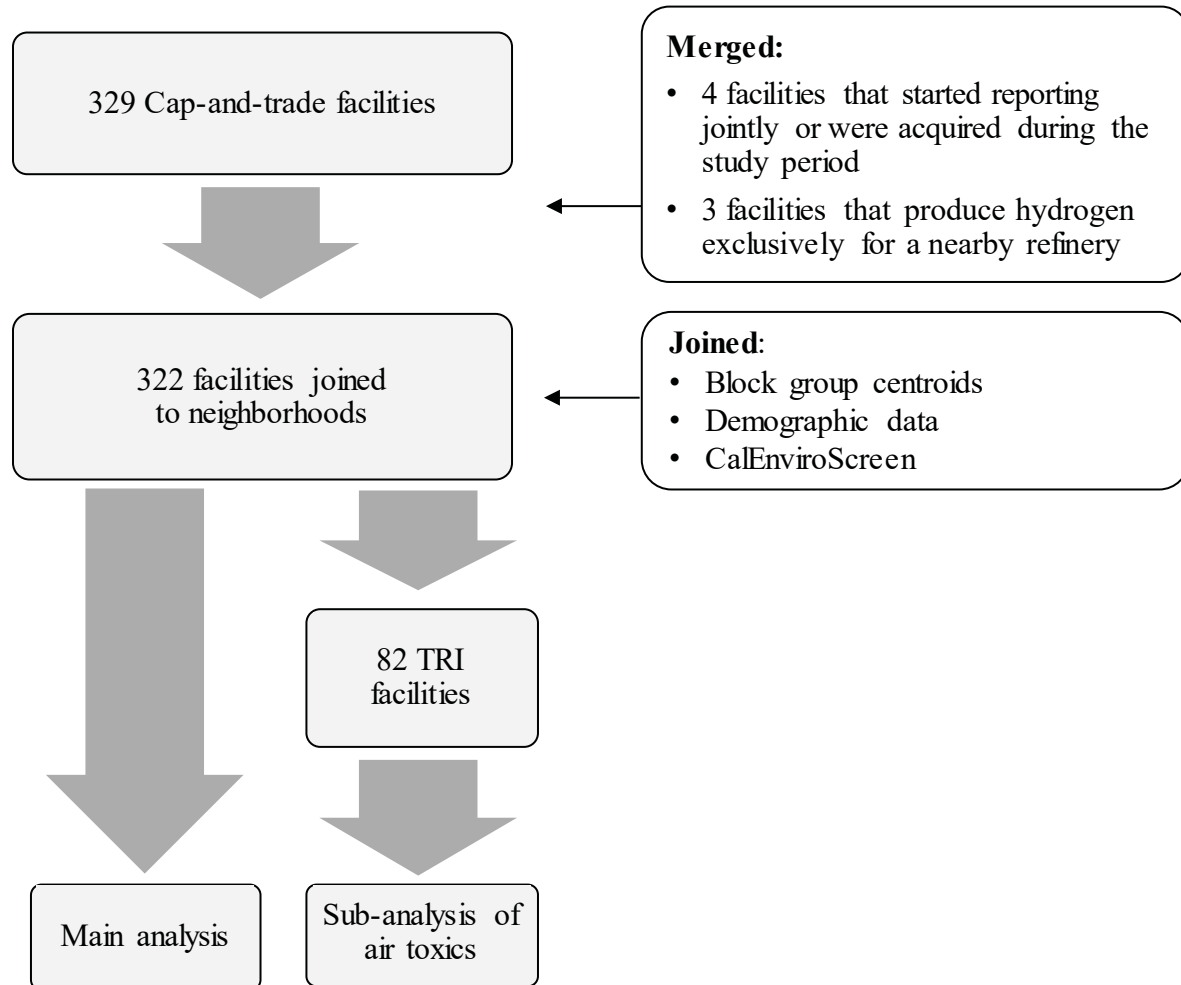

Supplement: S1 Fig — (PDF) [file pmed.1002604.s003.pdf]
