# Supplementary material for: Carbon trading, co-pollutants, and environmental equity: Evidence from California’s cap-and-trade program (2011–2015)
Source: PLoS Med. 2018 Jul 10;15(7):e1002604. doi: 10.1371/journal.pmed.1002604 (PMC6038989; doi:10.1371/journal.pmed.1002604)
Supplement: S2 Fig — (PDF) [file pmed.1002604.s004.pdf]

Figure S2. GHG allowances and emissions covered under California's cap-and-trade program, 2011-2015.

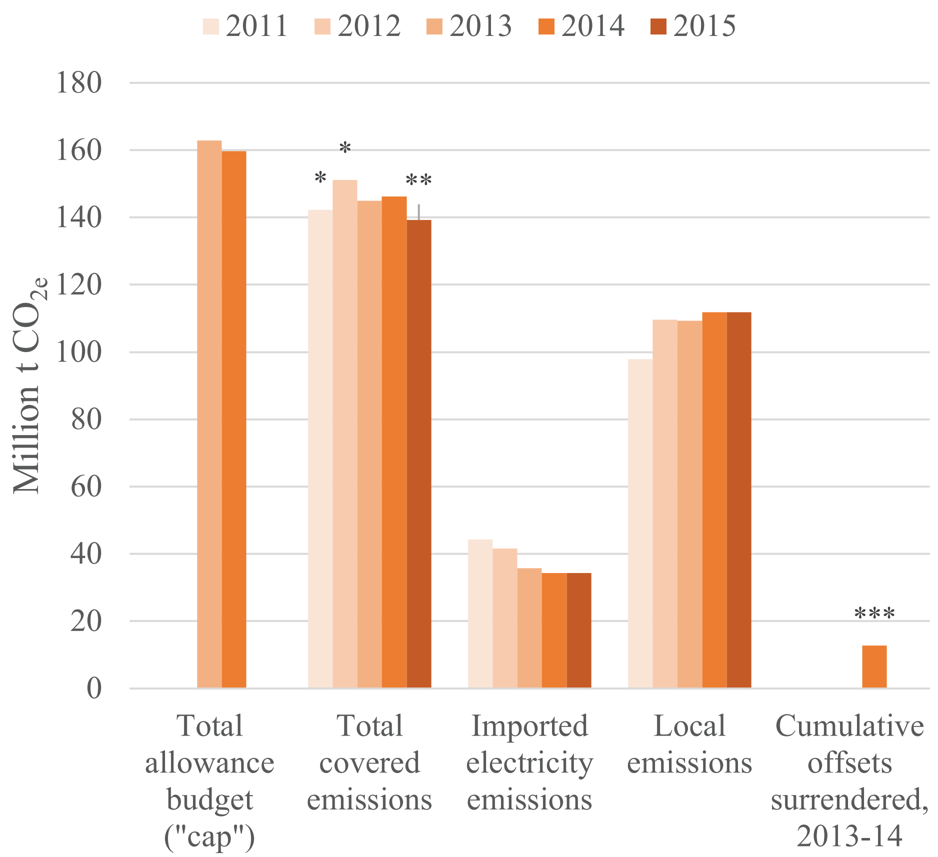

* Only 2013-15 emissions were subject to a compliance obligation. Estimates of comparable emissions during 2011 and 2012 were derived by summing the comparable local and electricity importer emissions reported by regulated facilities for those years.

** Transportation and natural gas fuel supplier emissions were also covered starting in 2015 but are omitted for comparability with prior years.

*** Total number of offsets surrendered during the first compliance period (2013-14).
