# Supplementary material for: Carbon trading, co-pollutants, and environmental equity: Evidence from California’s cap-and-trade program (2011–2015)
Source: PLoS Med. 2018 Jul 10;15(7):e1002604. doi: 10.1371/journal.pmed.1002604 (PMC6038989; doi:10.1371/journal.pmed.1002604)
Supplement: S1 Table — (PDF) [file pmed.1002604.s005.pdf]

Table S1: Characteristics of BGs within 1-mile of facilities regulated by California's cap-and-trade program.

	Within 1 miles of a facility (N = 1,490 BGs; N = 2,426,281people)	Beyond 1 miles of a facility (N = 21,700 BGs; N = 35,990,955 people)	P-value [a]
Median (IQR) population density (people/km^2)	3,297 (1540 – 6,505)	2,631 (1,212 – 4,487)	<0.001
Median (IQR) % people of color	67 (41 - 91)	58 (33 - 84)	<0.001
Median (IQR) % poor [b]	43 (23 - 62)	31 (16 - 51)	<0.001
Median (IQR) % low educational attainment [c]	20 (6 - 39)	13 (5 - 28)	<0.001
Median (IQR) % linguistically isolated households [d]	9 (3 - 19)	6 (1 - 14)	<0.001
% designated as a disadvantaged community [e]	44	23	<0.001†

[a] 2-Tailed Mann-Whitney-Wilcoxon test
[b] % of residents living below twice the federal poverty level
[c] % of residents older than 25 years without a high school education
[d] % of population living in households where no one >= age 14 speaks English very well
[e] Based on CalEnviroScreen 3.0 (see main text)
† Pearson's Chi-Squared test with Yates' Continuity Correctio
