# Supplementary material for: Carbon trading, co-pollutants, and environmental equity: Evidence from California’s cap-and-trade program (2011–2015)
Source: PLoS Med. 2018 Jul 10;15(7):e1002604. doi: 10.1371/journal.pmed.1002604 (PMC6038989; doi:10.1371/journal.pmed.1002604)
Supplement: S2 Table — (PDF) [file pmed.1002604.s006.pdf]

Table S2. Characteristics of BGs that experienced an increase in annual average aggregate emissions from regulated facilities within 1-mile post- (2013-15) as compared to pre- (2011-12) implementation of carbon trading.

	**GHGs decreased** (N = 770 BGs; 1,251,593 people)	**GHGs increased** (N = 464 BGs; 798,227 people)	**GHGs & PM2.5 increased** (N = 283 BGs; 481,233people)	**GHGs & NOx increased** (N = 291 BGs; 514,512 people)	**GHGs & SOx increased** (N = 238 BGs; 431,777 people)	**GHGs & VOCs increased** (N = 334 BGs; 575,713 people)	**GHGs & air toxics increased** (N = 97 BGs; 147,144 people)
Median (IQR) population density (people/km^2)	3,899 (2,031 – 7,651)	2,980*** (1,007 – 5,952)	2,861*** (854 - 6,017)	3,389*** (1,075 – 6,346)	1,934*** (597 – 3,719)	3,404*** (1,151 – 6,424)	3,921 (914 – 7,369)
Median (IQR) % people of color	66 (40 - 91)	75** (48 - 94)	85*** (6 - 96)	82*** (57 - 97)	75* (56 - 93)	81** (57 - 95)	90** (47 - 97)
Median (IQR) % poor	41 (21 - 63)	50*** (29 - 66)	51*** (29 - 68)	51*** (29 - 67)	49*** (30 - 67)	49** (28 - 67)	47 (23 - 65)
Median (IQR) % low education	18 (5 - 38)	25*** (11 - 46)	31*** (17 - 50)	29*** (15 - 48)	26*** (15 - 41)	25*** (11 - 47)	33** (10 - 50)
Median (IQR) % linguistically isolated	9 (3 - 20)	12* (4 - 21)	14*** (5 - 23)	13** (5 - 22)	12 (4 - 20)	13** (5 - 22)	13
% disadvantaged community	42	57***	66***	60***	58***	57***	68***

*** $p<0.001$, ** $p<0.01$, *$p<0.05$ compared to BGs where GHGs decreased, from Pearson's Chi-Squared test with Yates' Continuity Correction (for disadvantaged communities) or 2-Tailed Mann-Whitney-Wilcoxon test (all other variables)
