# Supplementary material for: Carbon trading, co-pollutants, and environmental equity: Evidence from California’s cap-and-trade program (2011–2015)
Source: PLoS Med. 2018 Jul 10;15(7):e1002604. doi: 10.1371/journal.pmed.1002604 (PMC6038989; doi:10.1371/journal.pmed.1002604)
Supplement: S3 Table — (PDF) [file pmed.1002604.s007.pdf]

Table S3. Differences in annual average air pollutant emissions from facilities regulated under California's cap-and-trade program by offset usage, 2013-14. Values are the median (interquartile range) of annual average emissions in metric tonnes.

	**Parent company used offsets (N= 198 facilities)**	**Parent company did not use offsets (N= 202 facilities)**	**P-value[a]**
Local GHGs	146,000 (57,700 – 571,000)	42,800 (27,200 – 82,900)	<0.001
$PM_{2.5}$	5.4 (2.0, 22.0)	4.2 (2.0, 11.4)	0.04
NO_x	21.3 (7.1 – 72.4)	16.7 (5.1 – 58.9)	0.18
SO_x	0.9 (0.3, 4.4)	0.6 (0.2, 4.0)	0.21
VOCs	2.3 (0.8, 9.1)	4.2 (1.1, 17.4)	0.002
Air toxics	1.3 (0.01 – 18.6)	0.4 (0.02 – 5.3)	0.40

[a] 2-Tailed Mann-Whitney-Wilcoxon test
