# Supplementary material for: Carbon trading, co-pollutants, and environmental equity: Evidence from California’s cap-and-trade program (2011–2015)
Source: PLoS Med. 2018 Jul 10;15(7):e1002604. doi: 10.1371/journal.pmed.1002604 (PMC6038989; doi:10.1371/journal.pmed.1002604)
Supplement: S4 Table — (PDF) [file pmed.1002604.s008.pdf]

**Table S4. Changes in mean aggregate air pollutant emissions from all regulated facilities after the implementation of CA's cap and trade program in 2013**

| | Mean aggregate emissions pre- cap and trade (2011-12) (t) | Mean aggregate emissions post- cap and trade (2013-15) (t) | Change in mean aggregate emissions (t) | Percent of facilities that increased mean emissions |
|---|---|---|---|---|
| Local GHGs (n=322) | 103,014,829 | 109,788,499 | 6,773,670 | 52% |
| $PM_{2.5}$ (n=322) | 6,995 | 7,042 | 47 | 51% |
| $NO_x$ (n=322) | 34,156 | 32,060 | -2,097 | 46% |
| $SO_x$ (n=322) | 11,475 | 9,927 | -1,548 | 44% |
| VOCs (n=322) | 9,832 | 10,417 | 585 | 57% |
| Air toxics (n=82) | 1,052 | 1,239 | 187 | 52% |
